# Endogenous retroviruses of non-avian/mammalian vertebrates illuminate diversity and deep history of retroviruses

**DOI:** 10.1371/journal.ppat.1007072

**Published:** 2018-06-14

**Authors:** Xiaoyu Xu, Huayao Zhao, Zhen Gong, Guan-Zhu Han

**Affiliations:** Jiangsu Key Laboratory for Microbes and Functional Genomics, Jiangsu Engineering and Technology Research Center for Microbiology, College of Life Sciences, Nanjing Normal University, Nanjing, Jiangsu, China; University of Utah, UNITED STATES

## Abstract

The deep history and early diversification of retroviruses remains elusive, largely because few retroviruses have been characterized in vertebrates other than mammals and birds. Endogenous retroviruses (ERVs) documented past retroviral infections and thus provide ‘molecular fossils’ for studying the deep history of retroviruses. Here we perform a comprehensive phylogenomic analysis of ERVs within the genomes of 92 non-avian/mammalian vertebrates, including 72 fishes, 4 amphibians, and 16 reptiles. We find that ERVs are present in all the genomes of jawed vertebrates, revealing the ubiquitous presence of ERVs in jawed vertebrates. We identify a total of >8,000 ERVs and reconstruct ~450 complete or partial ERV genomes, which dramatically expands the phylogenetic diversity of retroviruses and suggests that the diversity of exogenous retroviruses might be much underestimated in non-avian/mammalian vertebrates. Phylogenetic analyses show that retroviruses cluster into five major groups with different host distributions, providing important insights into the classification and diversification of retroviruses. Moreover, we find retroviruses mainly underwent frequent host switches in non-avian/mammalian vertebrates, with exception of spumavirus-related viruses that codiverged with their ray-finned fish hosts. Interestingly, ray-finned fishes and turtles appear to serve as unappreciated hubs for the transmission of retroviruses. Finally, we find retroviruses underwent many independent water-land transmissions, indicating the water-land interface is not a strict barrier for retrovirus transmission. Our analyses provide unprecedented insights into and valuable resources for studying the diversification, key evolutionary transitions, and macroevolution of retroviruses.

## Author summary

Retroviruses infect a wide range of vertebrates and cause many diseases, such as AIDS and cancers. To date, retroviruses have been rarely characterized in vertebrates other than mammals and birds, impeding our understanding of the diversity and early evolution of retroviruses. Retroviruses can occasionally integrate into host genomes and become endogenous retroviruses (ERVs), which provide molecular fossils for studying the long-term evolution of retroviruses. Here we performed comparative genomic and evolutionary analyses of ERVs within 92 non-avian/mammalian vertebrates (fishes, amphibians, and reptiles) and uncovered extraordinary diversity of retroviruses in non-avian/mammalian vertebrates. Our analyses reveal an ancient aquatic origin of retroviruses and retroviruses underwent frequent host-switching. Our findings have important implications in understanding the deep history and evolutionary mode of retroviruses.

## Introduction

Retroviruses (family *Retroviridae*) exclusively infect vertebrates and cause a wide variety of diseases, such as AIDS and cancers [1, 2]. Different from other RNA viruses, the replication of retroviruses requires reverse transcription of viral RNA into DNA and integration of the newly synthesized DNA into host chromosomes [1–3]. Retroviral infection primarily occurs in host somatic cells. On occasion, retroviruses infect germline cells, and the integrated retroviruses in germline cells (known as endogenous retroviruses [ERVs]) begin to be vertically inherited [1–3]. ERVs are thought to be highly abundant in the vertebrate genomes; for example, ERVs make up ~8% of the human genome [2]. Once embedded in host genomes, ERVs accumulate substitutions at a rate several orders of magnitude lower than exogenous retroviruses [3]. ERVs recorded past retroviral infections over time, sampling ancient extinct retroviral diversity. ERVs could thus provide ‘molecular fossils’ for studying the deep history and macroevolution of retroviruses as well as the host-retrovirus relationship [3–5].

Exogenous retroviruses are traditionally classified into seven genera, i.e. *Alpharetrovirus*, *Betaretrovirus*, *Gammaretrovirus*, *Deltaretrovirus*, *Epsilonretrovirus*, *Lentivirus*, and *Spumavirus* (also known as foamy virus), whereas ERVs do not follow the classification of exogenous retroviruses [3, 6]. Based on their relationships with exogenous retroviruses, ERVs are roughly classified into three classes: class I ERVs are closely related to gammaretroviruses and epsilonretroviruses, class II ERVs are closely related to betaretroviruses, and class III ERVs are closely related to foamy viruses [3, 7]. However, the ERV classification system has not been well designed and has many practical problems: i) the term “Class” ranks above the term “Family” in traditional taxonomy [3, 7]; ii) the classification systems for exogenous retroviruses and ERVs were developed separately and have been poorly incorporated; iii) some ERVs arose from recent endogenization events and nest within the diversity of exogenous retroviruses, such as endogenous lentiviruses identified recently in mammals [8–11], thus those ERVs cannot be readily classified into a certain ERV class.

The recent explosion of genome-scale data provides great opportunities to systemically analyze the diversity and evolution of ERVs within the vertebrate genomes. Multi-species genome-wide ERV studies have placed much emphasis on mammals and birds and unmasked many novel aspects of the distribution, diversity, and evolution of retroviruses [12–16]. However, many important issues related to early diversification, key evolutionary transitions, and macroevolutionary patterns of retroviruses remain to be clarified. Retroviral fossils within non-avian/mammalian vertebrates appear to hold the key to understanding the deep history and early diversification of retroviruses [17]. For example, the identification of endogenous foamy virus in fishes reveals an ancient marine origin of this retroviral group, and possibly the whole retroviruses [10, 18, 19]. Several attempts to mine ERVs in some non-avian/mammalian vertebrate genomes have been made [16, 20–22], but these genome-scale surveys exploited only very limited number of species (one to around ten).

Here we performed genome-wide mining of ERVs within the genomes of 92 non-avian/mammalian vertebrate species (72 fishes, 4 amphibians, and 16 reptiles), which include all the currently available genomes of non-avian/mammalian vertebrates. Analyses of ERVs within non-avian/mammalian vertebrates reveal unexpected retroviral diversity and clarify many issues in the classification, early diversification, key evolutionary transitions, and macroevolution of retroviruses.

## Results and discussion

### Mining ERVs in non-avian/mammalian vertebrates

To explore the diversity of ERVs in non-avian/mammalian vertebrates, we used a combined similarity search and phylogenetic analysis approach to identify ERVs in the genomes of non-avian/mammalian vertebrates. Briefly, we first performed similarity search to identify retrovirus-like sequences. Because retroviruses share detectable sequence similarity with other retrotransposons, we then performed phylogenetic analyses to identify authentic ERVs (see Materials and Methods for details). Our study includes a total of 92 non-avian/mammalian vertebrate species, including 2 jawless fishes, 3 cartilaginous fishes, 66 ray-finned fishes, 1 lobe-finned fish, 4 amphibians, and 16 reptiles (S1 Fig and S1 Table). These species include all the non-avian/mammalian vertebrates whose genomes have been sequenced to date and cover a broad range of non-avian/mammalian vertebrate diversity. Our ERV detection approach does not rely on identification of long terminal repeats (LTRs) first and is thus more sensitive for the detection of degraded or fragmented ERVs.

**Fig 1:**
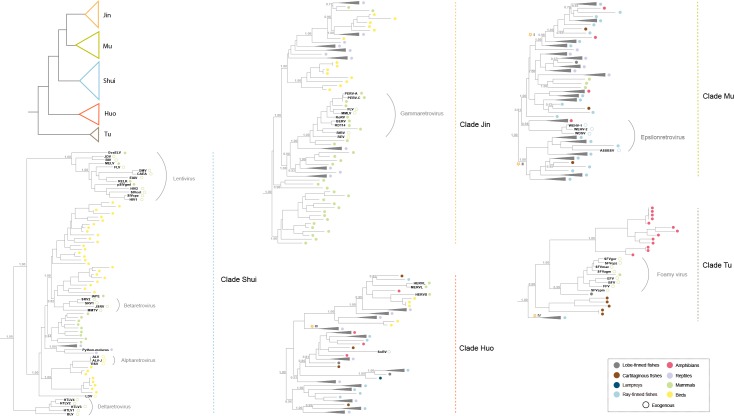
Phylogenetic relationship of non-avian/mammalian vertebrate ERVs, representative mammalian and avian ERVs, and exogenous retroviruses. The phylogenetic tree was reconstructed based on the RT protein and by using a maximum likelihood method. The numbers near the selected nodes indicate the aBayes branch supports. Selected retroviruses are labelled near the corresponding external nodes. The hollow circles indicate exogenous retroviruses, whereas the filled circles indicate ERVs. The root was inferred by using Cer1-6 retrotransposons as outgroups. For virus abbreviation, see S1 Table. For lineage I to IV with asterisks, we performed host-retrovirus co-phylogenetic tests in Fig 3 and Table 1.

We found the presence of ERVs in the genomes of all the jawed vertebrates, revealing the ubiquitous presence of ERVs in the genomes of jawed vertebrates [23]. Taken together, a total of 8,075 ERVs were identified (S1 Table; S1 Data). For jawless fishes, our genome-scale mining identified the presence of ERVs in the sea lamprey (*Petromyzon marinus*) but not in the Arctic lamprey (*Lethenteron camtschaticum*). ERVs were estimated to invade into lamprey genomes around 27–34 million years ago, which appears to occur after the divergence of the sea lamprey and the Arctic lamprey around 30–38 million years ago [24] and is compatible with the identification of ERVs in the sea lamprey but not the Arctic lamprey. The ERVs we identified in the sea lamprey are phylogenetically close to ERVs of ray-finned and lobe-finned fishes, indicating that the sea lamprey retrovirus might not represent an ancient retroviral lineage but might arise from a more recent cross-species transmission (Fig 1 and S2 Fig). We did not find any ERV within the genome of lancelet (*Branchiostoma floridae*), which belongs to subphylum Cephalochordata and is closely related to the subphylum Vertebrata. The distribution of ERVs in vertebrates implies that retroviruses originated within the vertebrate lineages, likely before the origin of jawed vertebrates >450 million years ago [25]. However, the possibility that retroviruses arose before the emergence of vertebrates and failed to colonize the germline of earlier-branching animals cannot be fully excluded. However, no jawed vertebrate species escaped the activity of ERVs, suggesting the high capability of endogenization of retroviruses and making the possibility of failing to colonize earlier-branching animal genomes highly unlikely.

### Phylogenetic diversity of retroviruses

We reconstructed 452 consensus sequences of partial or complete ERV genomes (S2 Data), because many ERVs identified here were highly degraded/fragmented and might confound phylogenetic and evolutionary analyses. To clarify the relationship among and evolutionary history of retroviruses, we performed phylogenetic analysis of the reconstructed non-avian/mammalian vertebrate ERVs, representative bird and mammal ERVs, and representative exogenous retroviruses (S2 Table). Our phylogenetic analysis recapitulates the conventional groupings of seven exogenous retroviral genera (Fig 1 and S2 Fig). Interestingly, exogenous retroviruses appear to only represent a small fraction of retroviral diversity (Fig 1 and S2 Fig). There are an enormous number of lineages dispersed outside the diversity of exogenous retroviral groups and ERVs of mammals and birds (Fig 1 and S2 Fig), suggesting the existence of extraordinary hidden diversity of retroviral diversity in non-avian/mammalian vertebrates. It is highly likely that there might be many uncharacterized exogenous retroviruses circulating among non-avian/mammalian vertebrates. Indeed, recent virus discovery studies based on meta-genomics and meta-transcriptomic approaches found many novel RNA viruses in non-avian/mammalian vertebrates [26, 27].

**Fig 2:**
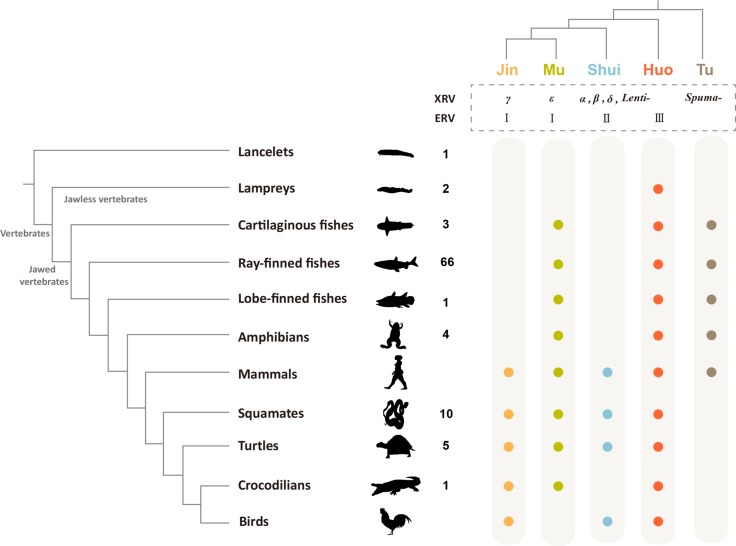
The distribution of major retroviral clades in vertebrates. The left panel shows the phylogenetic relationship among major vertebrate groups. The numbers near the vertebrates indicate the numbers of genomes used in this study. The top-right panel shows the phylogenetic relationship among the five major retroviral groups. XRV and ERV stand for exogenous and endogenous retrovirus, respectively. *α*, *β*, *γ*, *δ*, *ε*, *Lenti-*, and *Spuma-* represent *Alpharetrovirus*, *Betaretrovirus*, *Gammaretrovirus*, *Deltaretrovirus*, *Epsilonretrovirus*, *Lentivirus*, and *Spumavirus*, respectively. I, II, and III represent class I, II, and III ERVs. The filled circles indicate the presence of ERVs.

Our phylogenetic analysis shows that retroviruses group into five major clades with strong supports (Fig 1 and S2 Fig), which are designated clades Jin, Mu, Shui, Huo, and Tu, following *Wu Xing* (Five Elements) that traditional Chinese culture used to explain myriad of phenomena, from nature to medicine to politics. Clade Jin includes gammaretroviruses and exclusively infects amniotes. Clade Mu includes epsilonretroviruses and their hosts include nearly all the jawed vertebrates (except birds). Clade Shui is closely related to alpha-, beta-, delta-retroviruses and lentiviruses and infects amniotes. Clade Huo is related to snakehead retrovirus and has the widest host distribution, infecting all the major vertebrate lineages. Clade Tu is related to foamy viruses and has patchy host distributions; it infects jawed vertebrates but no Tu retrovirus has been found in reptiles and birds. It follows that different viral clades appear to have different host distributions (Fig 2). In terms of the relationship between major retroviral clades and ERV classes, Jin and Mu clades include class I ERVs, Clade Shui includes class II ERVs, and Clade Huo includes class III ERVs [17]. While the current classification systems of ERVs and exogenous retroviruses consider ERVs and exogenous retroviruses independently, our provisional nomenclature takes both exogenous and endogenous retroviruses into account. Nevertheless, the non-avian/mammalian vertebrate ERVs will provide a useful resource for further development of evolutionary history-based classification and nomenclature system of retroviruses.

### Origin of major retroviral groups

All the major jawed vertebrate groups are infected by viruses of at least three clades, whereas jawless fishes contain a single viral lineage within clade Huo. Among all the vertebrate groups, mammals are infected by all the five major lineages and have the widest viral spectrum (Fig 2). Fish retroviruses branch earlier than others within clades Mu, Huo, and Tu, indicating that these clades might have an aquatic origin. However, mammal retroviruses appear to occupy phylogenetically basal positions within clades Jin and Shui, and other amniote retroviruses fall within the diversity of mammal retroviruses. This phylogenetic pattern suggests that the current Jin and Shui retroviral diversity have a mammalian origin. However, it remains unclear how mammals were infected by Jin and Shui retroviruses. It is likely that there are Jin and Shui ERVs in non-avian/mammalian vertebrates and further genome-mining of non-avian/mammalian vertebrates might help solve these mysteries.

Our phylogenetic analysis has important implications in clarifying the origin and host distribution of many specific retroviral lineages: i) Fish epsilon-like retroviruses (clade Mu) were proposed to arise from multiple cross-species transmission events, possibly from amphibians [15]. However, this conclusion is based on screening limited number of non-avian/mammalian vertebrates. Our phylogenetic analysis shows that epsilon-like retroviruses originated in fish species and amphibian viruses arose multiple times through cross-species transmissions from fishes (Fig 1). Mammalian epsilon-like retroviruses fall into the diversity of reptile retroviruses and thus might originate from cross-species transmission from reptiles. ii) Mammalian gammaretroviruses (clade Jin) nest within reptile retroviruses, suggesting they arose from host-switching from reptiles to mammals. iii) ERVs related to betaretroviruses (within clade Shui) were found to be present in pythons. We also identified similar ERVs within the genomes of other Squamata species as well as Testudines, suggesting this retroviral lineage might be more widely distributed in reptiles.

### Cross-species transmission of retroviruses

Our phylogenetic analysis shows that retroviruses (except clade Tu) generally do not reflect the phylogenetic relationships of their hosts and retroviruses from distinct vertebrate groups are often closely related (Fig 1). For example, retroviruses of cartilaginous fishes do not occupy basal positions within any major retroviral clades, but were distributed throughout the phylogenetic tree. The phylogenetic pattern indicates retroviruses underwent complex and frequent host switches.

**Fig 3:**
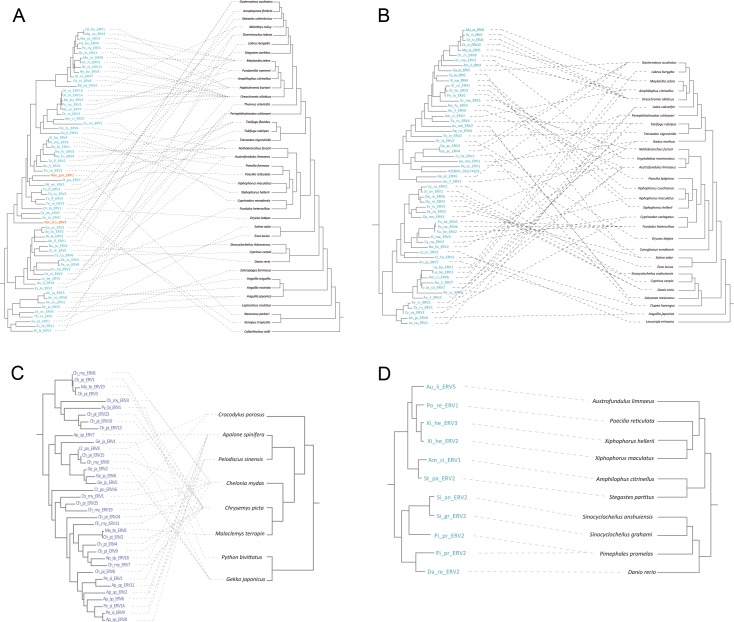
The host-retrovirus co-phylogenetic relationship in non-avian/mammalian vertebrates. (**A**) to (**D**) correspond to lineages I to IV with asterisks in Fig 1. For (**A**) to (**D**), the left and right panels represent retrovirus and host phylogenies, respectively. The dash lines indicate host-retrovirus association. Retroviruses of fishes, amphibians, and reptiles are labeled in blue, orange, and purple, respectively.

To estimate the relative importance of host switch and co-speciation in the evolution of non-avian/mammalian vertebrate retroviruses, we performed a global assessment of the correspondence between retrovirus and non-avian/mammalian vertebrate phylogenies using an event-based approach. Sampling bias might have important effects on the interpretation of host-virus relationship, as exemplified by primates and lentiviruses [28]. Because sampling of fishes (72 species) and reptiles (16 species) are relatively good in our study, we examined host-virus relationship for two fish retrovirus groups within clade Mu, one fish retrovirus group within clade Tu, and one reptile retrovirus group within clade Huo. Our analyses show that all these three retroviral lineages within clades Mu and Huo mainly underwent cross-species transmission (*p* > 0.05) (Fig 3A–3C and Table 1). However, the fish retroviruses within clade Tu (related to foamy virus) mainly co-diverged with their hosts (*p* < 0.01) (Fig 3D and Table 1). Indeed, foamy virus, which belongs to clade Tu, has been proposed to co-diverge with their hosts [10, 19, 29]. The reasons why the pattern of cross-species transmission for these retroviral groups are different remain largely unknown.

**Table 1:** Host-virus phylogeny congruence test for retroviruses.

Test^1^	Event costs^2^	Total cost	Cospeciation^3^	Duplication^3^	Duplication & host switching^3^	Loss^3^	Failure to diverge^3^	*P*-value^4^
I	-1,0,0,0,0	-13	13–13	9–9	14–14	12–15	0	>0.05
	0,1,1,2,0	29	7–7	8–8	21–21	0	0	>0.05
	0,1,2,1,1	47	8–9	12–13	15–15	4–5	0	>0.05
II	-1,0,0,0,0	-25	25–25	9–14	25–30	69–104	2–2	>0.05
	0,1,1,2,0	98	14–14	9–9	41–41	24–24	2–2	>0.05
	0,1,2,1,1	114	14–17	9–12	36–38	24–28	2–2	>0.05
III	-1,0,0,0,0	-13	13–13	9–9	14–14	12–15	0	>0.05
	0,1,1,2,0	29	7–7	8–8	21–21	0	0	>0.05
	0,1,2,1,1	47	8–9	12–13	15–15	4–5	0	>0.05
IV	-1,0,0,0,0	-9	9–9	0	1–1	0	0	<0.01
	0,1,1,2,0	1	9–9	0	1–1	0	0	<0.01
	0,1,2,1,1	2	9–9	0	1–1	0	0	<0.01

^1^Tests I-IV correspond to asterisks I-IV in Fig 1.

^2^Event cost schemes used in this study are for cospeciation, duplication, duplication & host switching, loss, failure to diverge, respectively.

^3^Numbers of events with the same total cost are expressed as ranges.

^4^*P*-value represents statistical test results by using random parasite tree algorithm with sample size of 500.

**Fig 4:**
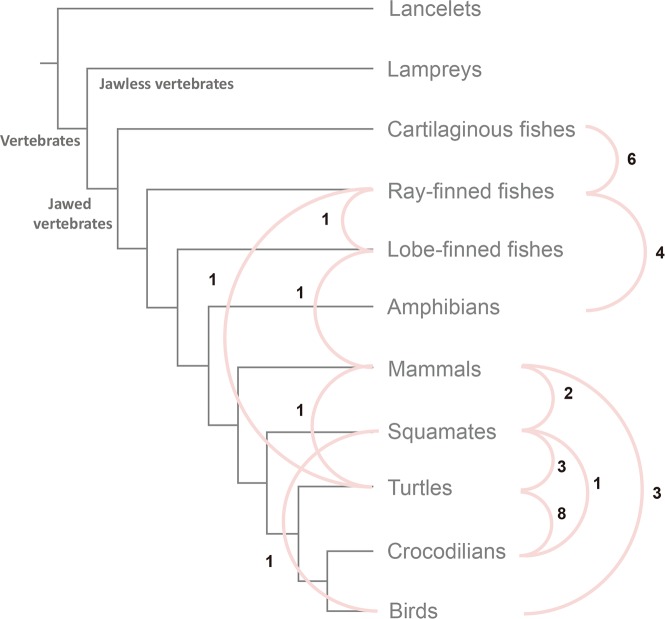
Transmission network of retroviruses among major vertebrate lineages. The gray lines represent the phylogenetic relationship among major vertebrate groups. The pink lines indicate retroviruses from two vertebrate groups share common ancestry at terminal nodes, which represent transmission events between hosts without known direction. The numbers show the frequencies of the corresponding transmission events.

Interclass transmission was thought to occur infrequently during the evolution of retroviruses, with only a few cases documented [14, 30, 31]; for example, avian reticuloendotheliosis viruses derived directly from mammalian retroviruses [31]. To further explore the transmission among major lineages of vertebrates, we reconstructed an undirected network in which edges represent transmission events between hosts without known direction (see Materials and Methods for details). Because the host states for most internal nodes cannot be reconstructed unambiguously, we only examine transmission events at terminal nodes, which might only reflect recent transmission events. We found that ray-finned fishes and turtles represent transmission “hubs”, which have high connectivity (12 and 13 transmission events, respectively) with other lineages (Fig 4). Transmission is more likely to occur between lineages with overlapping ecological niches; all the transmission partners of the ray-finned fishes live at least partially in aquatic environments. The number of interclass transmission events should be much underestimated, because the transmissions at terminal nodes might only reflect recent transmissions and these interclass transmissions might occur through other intermediate hosts. It follows that interclass transmission might be more frequent than previously thought. It should be noted that our analysis might be confounded by different frequencies with which different retroviral lineages invaded host germ lines and rate of fixation in host populations. Nevertheless, our analyses do suggest that retroviruses (except the clade Tu retroviruses) underwent complex and frequent host switches.

### Transmission at water-land interface

**Fig 5:**
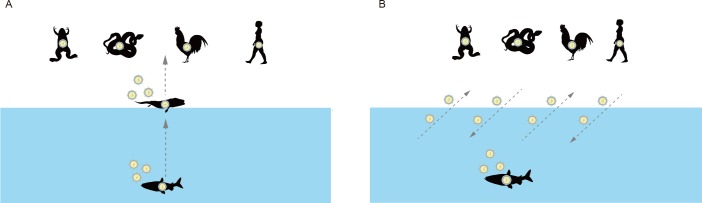
Retroviral transmission modes at the land-water interface. The blue boxes indicate aquatic environments. (**A**) Scenario where retroviruses underwent water-to-land transition simultaneously with the conquest of land by their tetrapod hosts. (**B**) Scenario where tetrapod retroviruses independently originated by cross-species transmissions from fishes to tetrapods after the origin of tetrapods.

It still remains unclear how retroviruses that infect tetrapods originated. There are two possible evolutionary scenarios: i) The retroviruses underwent water-to-land transition simultaneously with the conquest of land by their tetrapod hosts (Fig 5A); ii) The tetrapod retroviruses independently originated by cross-species transmissions from fishes to tetrapods after the origin of tetrapods (Fig 5B). Through the comprehensive phylogenetic analysis of retroviruses, we found retroviruses of aquatic and terrestrial origins are frequently interconnected with each other especially in clades Mu and Huo (S3 Fig), indicating many independent transfers between water and land. These transfers usually occurred among different vertebrate groups and do not have a common pattern, suggesting tetrapod retroviruses have multiple aquatic origins (Fig 1 and S3 Fig). For example, amphibian retroviruses within clade Mu nest within ray-finned fish viruses, which can be explained by recent cross-species transmission (Fig 1). Together with recent identification of several instances of cross-species transmission from aquatic to terrestrial vertebrates, such as hepadnaviruses [32, 33], our results suggest that the water-land interface might be not a strict barrier for the transmission of retroviruses.

## Conclusions

Previous multispecies studies have placed much emphasis on ERVs within the genomes of mammals [14, 16]. The studies on ERVs in non-avian/mammalian vertebrates, which account for >75% vertebrate diversity [34], involved only limited number of species (from one to about ten) [16, 20–22]. Here we perform a phylogenomic analysis of ERVs in 92 non-avian/mammalian vertebrates, representing the most comprehensive analysis of ERVs in non-avian/mammalian vertebrates. We provide a more sensitive workflow for identifying fragmented and degraded ERVs. Our analyses reveal the unappreciated diversity of retroviruses in non-avian/mammalian vertebrates and provide novel insights into the macroevolution and evolution of retroviruses in vertebrates. However, the non-avian/mammalian vertebrates we used in this study only represent a small proportion (~0.2%) of their extant diversity. Therefore, there are more endogenous retroviruses waiting for discovery, which might improve our understanding of the diversity and evolution of retroviruses. Understanding the diversity and evolution of retroviruses has important implications in helping predict further retroviral outbreaks and design control measures.

## Materials and methods

### ERV mining

All the genome sequences of non-avian/mammalian vertebrates were retrieved from NCBI genome resource (https://www.ncbi.nlm.nih.gov/genome/). Given retroviruses have coexisted with their vertebrate hosts for millions of years, some ancient ERVs might be fragmented and highly degraded. However, most of the automatic ERV detection software, such as RetroTector, are not tailored for the absence of LTRs and fail to detect the evolutionarily old ERVs [35]. Therefore, we used a combined similarity search and phylogenetic analysis approach to mine ERVs. First, we performed similarity search against the genomes of non-avian/mammalian vertebrates using tblastn algorithm with the Pol protein sequences of representative retroviruses as queries. Because there are many frameshift mutations within ERVs, many significant hits only correspond to partial regions of ERVs. We retrieved and concatenated the significant hits from tblastn results, if they are adjacent to each other in both ERV genome and host genome sequences. Next, because retroviruses share detectable sequence similarity with other retrotransposons, we performed phylogenetic analyses of concatenated sequences and sequences of representative retroviruses and retrotransposons [36]. The concatenated sequences that cluster with retroviruses are ERV sequences. The phylogenetic analyses were performed by an approximately maximum likelihood method implemented in FastTree 2.0 [37].

### Consensus sequence reconstruction

Given some recovered ERVs are fragmented, we reconstructed consensus sequences for ERVs. For the ERV cluster that contains sequences from one species in the phylogenetic tree based on the Pol proteins, we retrieved the longest ERV sequence within the ERV cluster. Then the ERV sequence was further used as a query to search its paralogous sequences within the same genome through the blastn algorithm with an *e* cutoff value of 10^−10^. Only the resulting significant hits within the 5,000 bp before/after the Pol proteins that belong to the ERV cluster were used to reconstruct consensus sequences of each retroviral cluster using Geneious 10 [38]. For the ERV cluster that contains sequences from two species, we reconstructed consensus sequence for each species. Conserved domains were identified by Conserved Domain Database (CDD) search [39].

### Phylogenetic analysis

All protein sequences were aligned using MAFFT version 7 with the E-INS-i strategy, an accurate method [40]. The alignment was then manually edited to remove ambiguous regions. We reconstructed phylogenetic tree based on the RT protein of the reconstructed consensus sequences of non-avian/mammalian vertebrate ERV and representative exogenous retroviruses and endogenous retroviruses (S2 Table). We used the RT protein sequences of Cer1-6 as outgroups, because Cer1-6 belong to the *Metaviridae* family and *Metaviridae* is the retrotransposon group most closely related to retroviruses [41]. The phylogenetic analysis was performed using a maximum-likelihood based algorithm implemented in PhyML 3.1 [42]. The RtRev substitution model which is specific for RT-containing genes [43] was used, with four gamma-distributed rate categories. The NNI tree topology search algorithm was used. The tree branch supports were evaluated by the aBayes algorithm [42].

### Dating ERV invasion into lamprey genomes

The ERVs within the genome of the sea lamprey cluster together, suggesting they arose from a single invasion event. The divergence among the ERVs in the sea lamprey reflects the invasion time. We retrieved the sea lamprey ERVs and aligned them using MAFFT version 7 [40]. Pairwise genetic distance among the sea lamprey ERVs was calculated with Kimura two-parameter substitution model. The invasion time *t* = *d/*2*μ*, where *d* is the largest pairwise distance among ERVs, and *μ* is the neutral evolutionary rate of hosts and is about 1.9–2.4 × 10^−9^ substitutions per site per year [24].

### Co-speciation analysis

To investigate the major macroevolutionary mode of retroviruses, we used an event-based method through Jane 4 [44] to assess the relationships between host and retrovirus phylogenies. Jane mapped five events of virus phylogeny (cospeciation, duplication, duplication & host switching, loss and failure to diverge) onto the host tree and each event was assigned to a cost. A best mapping was sought by minimizing the total cost. Inferred from previous documents, we assigned three cost schemes (cospeciation-duplication-duplication & host switching-loss-failure to diverge) shown as follows, 0-1-2-1-1 (Jane’s default setting), -1-0-0-0-0 [19, 45], and 0-1-1-2-0 [44]. Then Jane performed statistical analyses to assess the host-virus phylogeny congruence by generating random parasite trees, with the sample size of 500.

### Transmission network reconstruction

We first collapsed all the ERVs that are from species that belong to the same class (from the same order for reptiles, given reptiles are paraphyletic) and clustered together into a group. Because the host states for internal nodes cannot be reconstructed unambiguously and assigned to a specific state with 100% certainty, we identified two groups that share common ancestry at terminal nodes and assigned one undirected interclass transmission event for each such node. This method is more likely to identify recent transmission events.

## Supporting information

S1 Fig The phylogenetic relationship of vertebrates used in this study. (PDF)

S2 Fig The full view of the phylogenetic tree of retroviruses in Fig 1. (PDF)

S3 Fig Evolution of retroviruses at the land-water interface. The phylogenetic tree is based on Fig 1. The tip labels are based on the living environments of hosts.
(PDF)

S1 Table Non-avian/mammalian vertebrate used in this study and ERVs identified. (PDF)

S2 Table The representative retroviruses used for phylogenetic analysis. (PDF)

S1 Data Information for the ERVs identified in this study. (TXT)

S2 Data The reconstructed ERV consensus sequences. (TXT)
