# Supplementary figures and images for: Endogenous retroviruses of non-avian/mammalian vertebrates illuminate diversity and deep history of retroviruses
Source: PLoS Pathog. 2018 Jun 14;14(6):e1007072. doi: 10.1371/journal.ppat.1007072 (PMC6001957; doi:10.1371/journal.ppat.1007072)

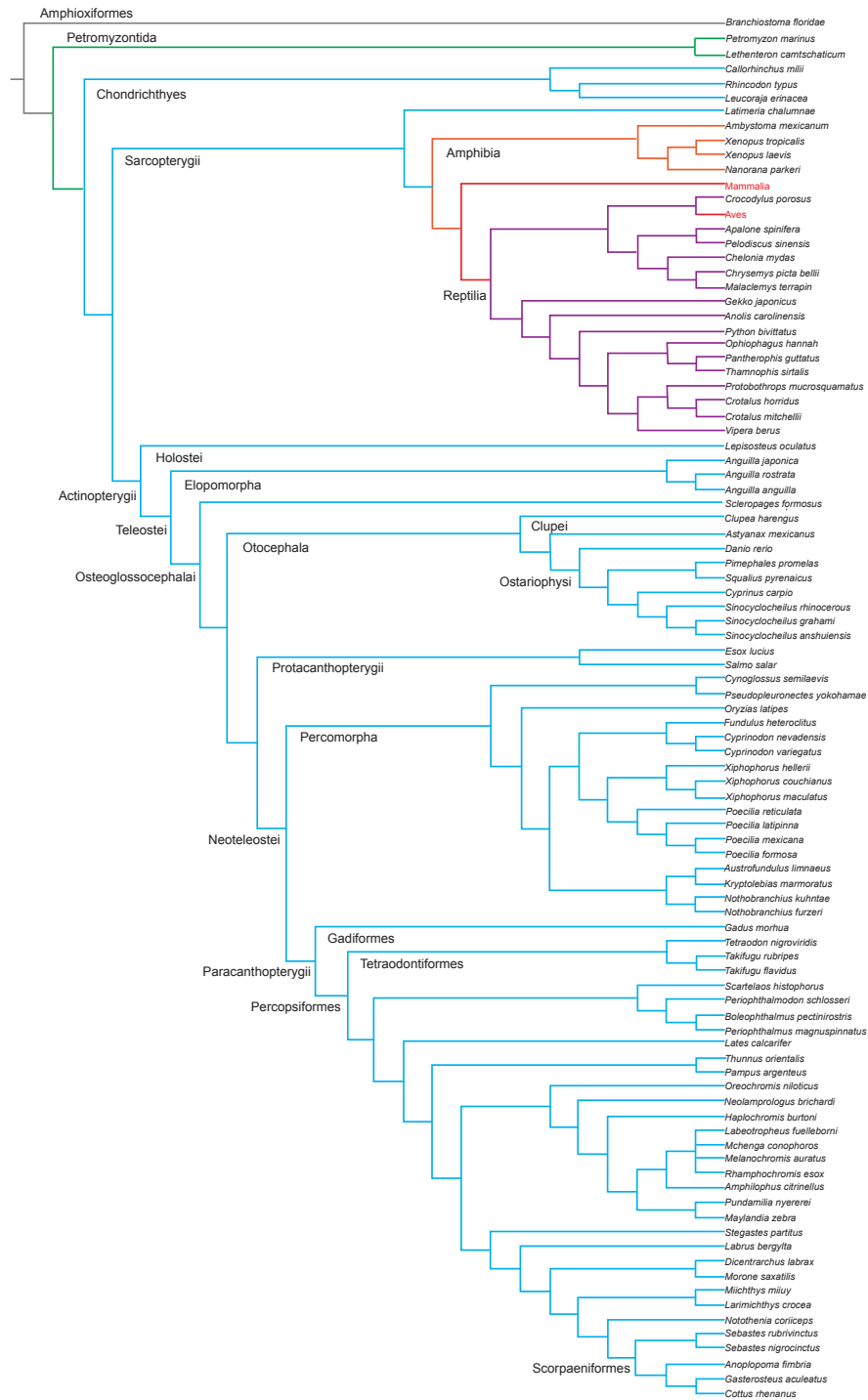

**S1 Fig. The phylogenetic relationship of vertebrates used in this study.**

Supplement: S1 Fig — (PDF) [file ppat.1007072.s001.pdf]

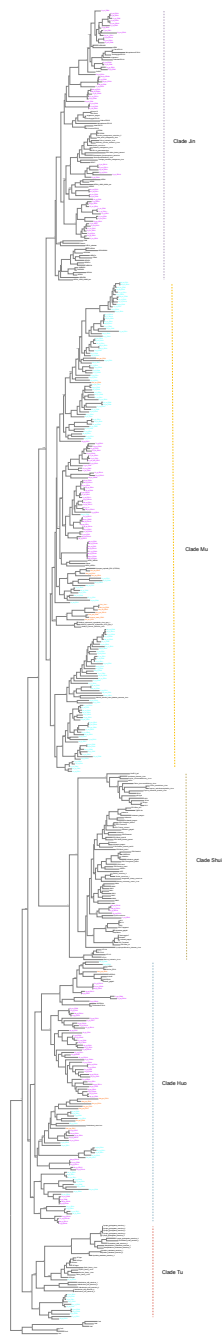

**S2 Fig. The full view of the phylogenetic tree of retroviruses in Figure 1.**

Supplement: S2 Fig — (PDF) [file ppat.1007072.s002.pdf]
