# Supplementary material for: Endogenous retroviruses of non-avian/mammalian vertebrates illuminate diversity and deep history of retroviruses
Source: PLoS Pathog. 2018 Jun 14;14(6):e1007072. doi: 10.1371/journal.ppat.1007072 (PMC6001957; doi:10.1371/journal.ppat.1007072)
Supplement: S3 Fig — The phylogenetic tree is based on Fig 1. The tip labels are based on the living environments of hosts. (PDF) [file ppat.1007072.s003.pdf]

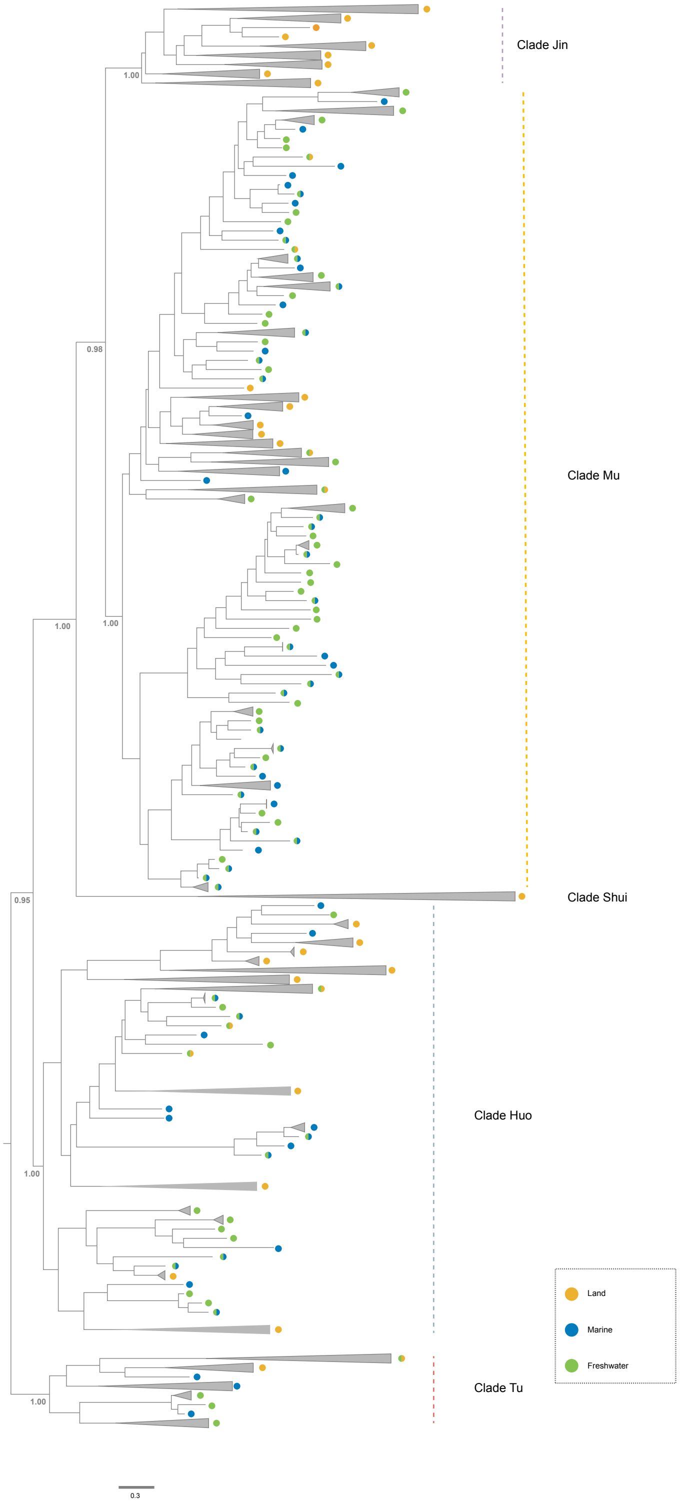

S3 Fig. Evolution of retroviruses at the land-water interface. The phylogenetic tree is based on Figure 1. The tip labels are based on the living environments of hosts.
