# Supplementary material for: Endogenous retroviruses of non-avian/mammalian vertebrates illuminate diversity and deep history of retroviruses
Source: PLoS Pathog. 2018 Jun 14;14(6):e1007072. doi: 10.1371/journal.ppat.1007072 (PMC6001957; doi:10.1371/journal.ppat.1007072)
Supplement: S1 Table — (PDF) [file ppat.1007072.s004.pdf]

S1 Table. Non-avian/mammalian vertebrate used in this study and ERVs identified

Species	Abbreviation	Class	Order	Size (Mb)	ERV No.	Consensus Sequence No.
Anguilla anguilla	An_an	Actinopterygii	Anguilliformes	1018.7	42	5
Anguilla japonica	An_ja	Actinopterygii	Anguilliformes	1151.14	55	7
Anguilla rostrata	An_ro	Actinopterygii	Anguilliformes	1413.05	54	7
Oryzias latipes	Or_la	Actinopterygii	Beloniformes	869.818	76	3
Astyanax mexicanus	As_me	Actinopterygii	Characiformes	1191.24	17	2
Clupea harengus	Cl_ha	Actinopterygii	Clupeiformes	807.712	27	4
Cyprinus carpio	Cy_ca	Actinopterygii	Cypriniformes	1713.64	173	7
Danio rerio	Da_re	Actinopterygii	Cypriniformes	1371.72	157	6
Pimephales promelas	Pi_pr	Actinopterygii	Cypriniformes	1219.33	17	2
Sinocyclocheilus anshuiensis	Si_an	Actinopterygii	Cypriniformes	1632.72	45	3
Sinocyclocheilus grahami	Si_gr	Actinopterygii	Cypriniformes	1750.29	49	4
Sinocyclocheilus rhinocerous	Si_rh	Actinopterygii	Cypriniformes	1655.79	36	1
Squalius pyrenaicus	Sq_py	Actinopterygii	Cypriniformes	48.1393	4	0
Austrofundulus limnaeus	Au_li	Actinopterygii	Cyprinodontiformes	866.963	147	8
Cyprinodon nevadensis	Cy_ne	Actinopterygii	Cyprinodontiformes	1011.85	18	1
Cyprinodon variegatus	Cy_va	Actinopterygii	Cyprinodontiformes	1035.18	61	4
Fundulus heteroclitus	Fu_he	Actinopterygii	Cyprinodontiformes	1021.9	84	6
Kryptolebias marmoratus	Kr_ma	Actinopterygii	Cyprinodontiformes	680.349	20	3
Nothobranchius furzeri	No_fu	Actinopterygii	Cyprinodontiformes	1242.52	86	6
Nothobranchius kuhntae	No_ku	Actinopterygii	Cyprinodontiformes	5.23461	10	2
Poecilia formosa	Po_fo	Actinopterygii	Cyprinodontiformes	748.923	19	1
Poecilia latipinna	Po_la	Actinopterygii	Cyprinodontiformes	815.145	10	1
Poecilia mexicana	Po_me	Actinopterygii	Cyprinodontiformes	801.711	9	0
Poecilia reticulata	Po_re	Actinopterygii	Cyprinodontiformes	731.622	28	2
Xiphophorus couchianus	Xi_co	Actinopterygii	Cyprinodontiformes	708.396	38	1
Xiphophorus hellerii	Xi_he	Actinopterygii	Cyprinodontiformes	733.802	68	5
Xiphophorus maculatus	Xi_ma	Actinopterygii	Cyprinodontiformes	729.664	47	5
Esox lucius	Es_lu	Actinopterygii	Esociformes	904.453	284	5
Gadus morhua	Ga_mo	Actinopterygii	Gadiformes	824.311	31	4
Lepisosteus oculatus	Le_oc	Actinopterygii	Lepisosteiformes	945.878	52	3
Scleropages formosus	Sc_fo	Actinopterygii	Osteoglossiformes	777.343	26	2

Amphilophus citrinellus	Am_ci	Actinopterygii	Perciformes	844.903	119	7
Boleophthalmus pectinirostris	Bo_pe	Actinopterygii	Perciformes	955.735	11	0
Dicentrarchus labrax	Di_la	Actinopterygii	Perciformes	675.917	41	3
Haplochromis burtoni	Ha_bu	Actinopterygii	Perciformes	831.412	56	5
Labeotropheus fuelleborni	La_fu	Actinopterygii	Perciformes	70.8584	16	2
Labrus bergylta	La_be	Actinopterygii	Perciformes	805.481	94	6
Larimichthys crocea	La_cr	Actinopterygii	Perciformes	648.407	34	0
Lates calcarifer	La_ca	Actinopterygii	Perciformes	668.465	47	2
Maylandia zebra	Ma_ze	Actinopterygii	Perciformes	859.842	101	8
Mchenga conophoros	Mc_co	Actinopterygii	Perciformes	73.4256	8	0
Melanochromis auratus	Me_au	Actinopterygii	Perciformes	68.2386	16	0
Neolamprologus brichardi	Ne_br	Actinopterygii	Perciformes	847.91	43	1
Notothenia coriiceps	No_co	Actinopterygii	Perciformes	636.614	2	1
Oreochromis niloticus	Or_ni	Actinopterygii	Perciformes	927.696	147	14
Pampus argenteus	Pa_ar	Actinopterygii	Perciformes	350.449	17	0
Periophthalmodon schlosseri	Pe_sc	Actinopterygii	Perciformes	679.761	49	6
Periophthalmus magnuspinnatus	Pe_ma	Actinopterygii	Perciformes	701.697	22	1
Pundamilia nyererei	Pu_ny	Actinopterygii	Perciformes	830.133	42	3
Rhamphochromis esox	Rh_es	Actinopterygii	Perciformes	71.2951	6	0
Scartelaos histophorus	Sc_hi	Actinopterygii	Perciformes	695.009	27	1
Stegastes partitus	St_pa	Actinopterygii	Perciformes	800.492	29	2
Thunnus orientalis	Th_or	Actinopterygii	Perciformes	684.497	48	4
Miichthys miiuy	Mi_mi	Actinopterygii	Perciformes	619.301	35	4
Morone saxatilis	Mo_sa	Actinopterygii	Perciformes	585.167	8	0
Gasterosteus aculeatus	Ga_ac	Actinopterygii	Gasterosteiformes	446.611	125	6
Anoplopoma fimbria	An_fi	Actinopterygii	Scorpaeniformes	699.326	26	2
Cottus rhenanus	Co_rh	Actinopterygii	Scorpaeniformes	563.609	5	0
Sebastes nigrocinctus	Se_ni	Actinopterygii	Scorpaeniformes	687.55	13	1
Sebastes rubrivinctus	Se_ru	Actinopterygii	Scorpaeniformes	756.297	18	1
Pseudopleuronectes yokohamae	Ps_yo	Actinopterygii	Pleuronectiformes	547.831	18	1
Cynoglossus semilaevis	Cy_se	Actinopterygii	Pleuronectiformes	470.199	28	2
Salmo salar	Sa_sa	Actinopterygii	Salmoniformes	2966.89	79	2
Takifugu flavidus	Ta_fl	Actinopterygii	Tetraodontiformes	378.032	82	4
Takifugu rubripes	Ta_ru	Actinopterygii	Tetraodontiformes	391.485	21	4
Tetraodon nigroviridis	Te_ni	Actinopterygii	Tetraodontiformes	342.403	48	3
Callorhinchus milii	Ca_mi	Chondrichthyes	Chimaeriformes	974.499	176	6

Leucoraja erinacea	Le_er	Chondrichthyes	Batoidea	1555.46	161	5
Rhincodon typus	Rh_ty	Chondrichthyes	Orectolobiformes	2567.63	349	4
Latimeria chalumnae	La_ch	Sarcopterygii	Coelacanthiformes	2860.59	177	5
Petromyzon marinus	Pet_mar	Petromyzontida	Petromyzontiformes	885.535	14	1
Branchiostoma floridae	Br_fl	Leptocardii	Amphioxiformes	N/A	0	0
Lethenteron camtschaticum	Le_ca	Petromyzontida	Petromyzontiformes	1030.66	0	0
Xenopus tropicalis	Xen_tro	Amphibia	Anura	1440.4	43	4
Xenopus laevis	Xen_lae	Amphibia	Anura	2718.43	78	7
Ambystoma mexicanum	Am_me	Amphibia	Urodela	353.835	1	0
Nanorana parkeri	Nan_par	Amphibia	Anura	2053.87	182	10
Crocodylus porosus	Cr_po	Reptilia	Crocodilia	2049.54	399	18
Apalone spinifera	Ap_sp	Reptilia	Testudines	1931.08	215	19
Chelonia mydas	Ch_my	Reptilia	Testudines	2208.41	320	19
Chrysemys picta bellii	Ch_pi	Reptilia	Testudines	2365.77	409	25
Malaclemys terrapin	Ma_te	Reptilia	Testudines	2439.75	704	29
Pelodiscus sinensis	Pe_si	Reptilia	Testudines	2202.48	198	16
Protobothrops mucrosquamatus	Pr_mu	Reptilia	Squamata	1673.88	337	11
Python bivittatus	Py_bi	Reptilia	Squamata	1435.05	65	6
Thamnophis sirtalis	Th_si	Reptilia	Squamata	1424.9	66	6
Vipera berus	Vi_be	Reptilia	Squamata	1532.39	56	6
Anolis carolinensis	An_ca	Reptilia	Squamata	1799.14	241	5
Pantherophis guttatus	Pa_gu	Reptilia	Squamata	1404.22	82	6
Ophiophagus hannah	Op_ha	Reptilia	Squamata	1594.07	49	6
Crotalus mitchellii	Cr_mi	Reptilia	Squamata	N/A	82	11
Crotalus horridus	Cr_ho	Reptilia	Squamata	1520.33	257	14
Gekko japonicus	Ge_ja	Reptilia	Squamata	2490.27	143	7
