# Supplementary material for: Endogenous retroviruses of non-avian/mammalian vertebrates illuminate diversity and deep history of retroviruses
Source: PLoS Pathog. 2018 Jun 14;14(6):e1007072. doi: 10.1371/journal.ppat.1007072 (PMC6001957; doi:10.1371/journal.ppat.1007072)
Supplement: S2 Table — (PDF) [file ppat.1007072.s005.pdf]

S2 Table. The representative retroviruses used for phylogenetic analysis

Virus Name	Accession No./Ref.
Bovine foamy virus (BFV)	NC 001831.1
Equine foamy virus (EFV)	NC_002201.1
Feline foamy virus (FFV)	NC_001871.1
Spider monkey foamy virus (SFVspm)	EU010385.1
Simian foamy virus-gorilla (SFVgor)	HM245790.1
simian foamy viruses of chimpanzees (SFVcpz)	NC_001364.1
Macaque simian foamy virus (SFVmac)	NC_010819.1
African green monkey simian foamy virus (SFVagm)	NC_010820.1
Bovine leukemia virus (BLV)	NP_056895.1
Human T-cell leukemia virus type 1 (HTLV-1)	NP_057860.1
Human T-cell leukemia virus type 2 (HTLV-2)	NP_041003.2
Human T-cell leukemia virus type 3 (HTLV-3)	DQ093792.1
Human T-cell leukemia virus type 4 (HTLV-4)	EF488483.1
Bovine immunodeficiency virus (BIV)	NP_040563.1
Human immunodeficiency virus type 2 (HIV-2)	NP_663784.1
Human immunodeficiency virus type 1 (HIV-1)	ABK51636.1
Simian immunodeficiency virus of colobus (SIVcol)	AAK01033.1
Simian immunodeficiency virus of chimpanzee (SIVcpz)	ABU53017.1
Jembrana disease virus (JDV)	AAA64389.1
Caprine arthritis-encephalitis virus (CAEV)	NP_040939.1
Ovine maedivisna virus (OMV)	YP_536867.1
Equine infectious anemia virus (EIAV)	NP_056902.1
Feline immunodeficiency virus (FIV)	NP_040973.1
Lymphoproliferative disease virus (LDV)	AAA62195.1
Avian leukosis virus (ALV)	YP_004222728.1
Avian leukosis virus subgroup J (ALV-J)	HQ900844.1
Rous sarcoma virus (RSV)	NP_056886.1
Murine endogenous retrovirus ERV-L (MERVL)	CAA73251.1
Python molurus endogenous retrovirus (Python-molurus)	AAN77283.1
Mouse mammary tumor virus (MMTV)	NP_056880.1
Jaagsiekte sheep retrovirus (JSRV)	NP_041186.1

Simian retrovirus 2 (SRV2)	AAA47562.1
Simian retrovirus 1 (SRV1)	AAA47732.1
Intracisternal A-type particle (IAPE)	Ref. 1
Snakehead retrovirus (SnRV)	NP_043924.1
Walleye dermal sarcoma virus (WDSV)	NP_045937.1
Walleye epidermal hyperplasia virus type 1 (WEHV-1)	AF133051.1
Walleye epidermal hyperplasia virus type 2 (WEHV-2)	AF133052.1
Atlantic salmon swim bladder sarcoma virus (ASSBSV)	NC_007654.1
Feline leukemia virus (FLV)	NP_955577.1
Moloney murine leukemia virus (MMLV)	NP_057933.2
Baboon endogenous virus (BERV)	BAA89659.1
Koala retrovirus (KoRV)	AF151794
RD114 retrovirus (RD114)	NC_009889.1
Reticuloendotheliosis virus (REV)	FJ439119.1
Rhinolophus ferrumequinum retrovirus (RfRV)	JQ303225.1
Gibbon ape leukemia virus	NP_056790.1
Sus scrofa endogenous virus (PERV-A)	EU789636.1
Porcine endogenous retrovirus C (PERV-C)	CAC39617.1
Xenopus tropicalis ERV (XTERV2)	HM765512
Rabbit endogenous lentivirus type K (RELIK)	Ref. 2
Gray mouse lemur prosimian immunodeficiency virus (pSIVgml)	Ref. 3
Sloth endogenous foamy virus	Ref. 4
Mustelidae endogenous lentivirus (MELV)	Ref. 5
Galeopterus variegatus endogenous lentivirus (GvaELV)	Ref. 6
Anas-ERV-R	Ref. 7
Columba-ERV-R	Ref. 7
Columba-ERV-H	Ref. 7
Falco-ERV-H	Ref. 7
Gallus-ERV-R	Ref. 7
Melopsittacus-ERV-H	Ref. 7
Melopsittacus-ERV-R	Ref. 7
ChiRV1	Ref. 7
RV-Rook	Ref. 7
Taeniopygia-ERV-H	Ref. 7

Taeniopygia-ERV-R	Ref. 7
Corvus-R	AJ236130.1
ERV-Asian_house_mouse-I	Ref. 1
ERV-Black_duck-I	AY820068.1
ERV-Black_duck-III	AY820070.1
ERV-Blue_tit-I	AY820077.1
ERV-Blue_tit-II	AY820078.1
ERV-Darwins_rhea-I	Ref. 1
ERV-Eastern_screech_owl-III	Ref. 1
ERV-Ferruginous_hawk-I	Ref. 1
ERV-Loon-I	AY820074.1
ERV-Ostrich-I	AY820094.1
ERV-Peacock-I	Ref. 1
ERV-Peregrine_falcon_I	Ref. 1
ERV-Tinamou-II	Ref. 1
ERV-Toucanet-I	Ref. 1
ERV-Tragopan-I	Ref. 1
ERV-Turkey_vulture-I	AY820062.1
GfERV1	Ref. 1
GGERV10	Ref. 1
GGERV29	Ref. 1
GGERVLA	Ref. 1
Taeniopygia-F	Ref. 1
TgERV-2a	Ref. 1
Zonotrichia-ERV-A	Ref. 1
Callorhinchus_milii-Retrovirus-1	AAVX02021479.1
Callorhinchus_milii-Retrovirus-2	AAVX02030290.1
Callorhinchus_milii-Retrovirus-3	AAVX02008076.1
Callorhinchus_milii-Retrovirus-4	AAVX02007855.1
Callorhinchus_milii-Retrovirus-5	XM007890932.1
Cynops_pyrrhogaster-Retrovirus-1	FS304312.1
Cynops_pyrrhogaster-Retrovirus-2	FS304159.1
Cynops_pyrrhogaster-Retrovirus-3	FS304248.1

Cynops_pyrrhogaster-Retrovirus-4	FS304221.1
Cynops_pyrrhogaster-Retrovirus-5	FS296312.1
Cynops_pyrrhogaster-Retrovirus-6	FS302975.1
Pleurodeles_waltl-Retrovirus-1	JG015238
Pleurodeles_waltl-Retrovirus-2	JG014957
Notophthalmus_viridescens-Retrovirus-1	JF490018.1
Notophthalmus_viridescens-Retrovirus-2	JF490018.1
Hynobius_retardatus-Retrovirus-1	LE148026.1
Hynobius_retardatus-Retrovirus-2	LE148029.1
Hynobius_retardatus-Retrovirus-3	LE148029.1
Alphabeta_galgal3	Ref. 8
Alphabeta	Ref. 8
Avibeta1a	Ref. 8
Avibeta1a_taegut1	Ref. 8
Avibeta1b	Ref. 8
Avibeta1b_taegut1	Ref. 8
Avibeta1c_galgal3	Ref. 8
Avibeta1c	Ref. 8
Avibeta1d	Ref. 8
Avibeta1d_taegut1	Ref. 8
Avibeta1e_galgal3	Ref. 8
Avibeta1e	Ref. 8
Avibeta2	Ref. 8
Avibeta2_taegut1	Ref. 8
Avierviii_galgal3	Ref. 8
AviervIII	Ref. 8
Avigamma1	Ref. 8
Avigamma1_taegut1	Ref. 8
HERV1	Ref. 9
HERV1_artiodact	Ref. 9
HERV3	Ref. 9
HERV4_10250_100014_con	Ref. 9

HERV4_10760_100005_con	Ref. 9
HERV9	Ref. 9
HERVADP	Ref. 9
HERVE	Ref. 9
HERVFa	Ref. 9
HERVFb	Ref. 9
HERVH48	Ref. 9
HERVL	Ref. 9
HERVS	Ref. 9
HERVT	Ref. 9
HML1	Ref. 9
HML2	Ref. 9
HML3	Ref. 9
HML4	Ref. 9
HML5	Ref. 9
HML6	Ref. 9
HML7	Ref. 9
HML8	Ref. 9
HML9	Ref. 9
HUERSP2	Ref. 9
HUERSP3	Ref. 9
LTR46	Ref. 9
PABL	Ref. 9
Prima4	Ref. 9
Prima41	Ref. 9
HERVFc1	Ref. 10
HERVFc2	Ref. 10
HERV_HEPSI3	Ref. 10
HERV_HEPSI1	Ref. 10
HERVHRGH2	Ref. 10
HERVI	Ref. 10
HERVW	Ref. 10

HERVH	Ref. 10
HERVFRD	Ref. 10

References

1. Gifford RJ. (2013). Retrovirus reference sequence library (version 2013-12-26). Link: http://bioinformatics.cvr.ac.uk/paleovirology/site/html/retroviruses.html
2. Katzourakis A, Tristem M, Pybus OG, Gifford RJ (2007). Discovery and analysis of the first endogenous lentivirus. Proc Natl Acad Sci USA 104:6261-6265.
3. Gifford RJ, Katzourakis A, Tristem M, Pybus OG, Winters M, Shafer RW. (2008). A transitional endogenous lentivirus from the genome of a basal primate and implications for lentivirus evolution. Proc Natl Acad Sci USA 105:20362-20367.
4. Katzourakis A, Gifford RJ, Tristem M, Gilbert MT, Pybus OG (2009). Macroevolution of complex retroviruses. Science 325:1512.
5. Han GZ, Worobey M. (2012). Endogenous lentiviral elements in the weasel family (Mustelidae). Mol Biol Evol 29:2905-2908.
6. Han GZ, Worobey M. (2015). A primitive endogenous lentivirus in a colugo: insights into the early evolution of lentiviruses. Mol Biol Evol 32:211-215.
7. Niewiadomska AM, Gifford RJ. (2013). The extraordinary evolutionary history of the reticuloendotheliosis viruses. PLoS Biol 11:e1001642.
8. Bolisetty M, Blomberg J, Benachenhou F, Sperber G, Beemon K. (2012). Unexpected diversity and expression of avian endogenous retroviruses. MBio 3:e00344-12.
9. Vargiu L, Rodriguez-Tomé P, Sperber GO, Cadeddu M, Grandi N, Blikstad V, Tramontano E, Blomberg J. (2016). Classification and characterization of human endogenous retroviruses; mosaic forms are common. Retrovirology 13:7.
10. Jern P, Sperber GO, Blomberg J. (2005). Use of endogenous retroviral sequences (ERVs) and structural markers for retroviral phylogenetic inference and taxonomy. Retrovirology 2:50.
